# Supplementary material for: Alternative molecular mechanisms for force transmission at adherens junctions via β-catenin-vinculin interaction
Source: Nat Commun. 2024 Jul 5;15:5608. doi: 10.1038/s41467-024-49850-5 (PMC11226457; doi:10.1038/s41467-024-49850-5)
Supplement: Supplementary file 1 — Supplementary Information File [file 41467_2024_49850_MOESM1_ESM.pdf]

SUPPLEMENTARY FIGURES

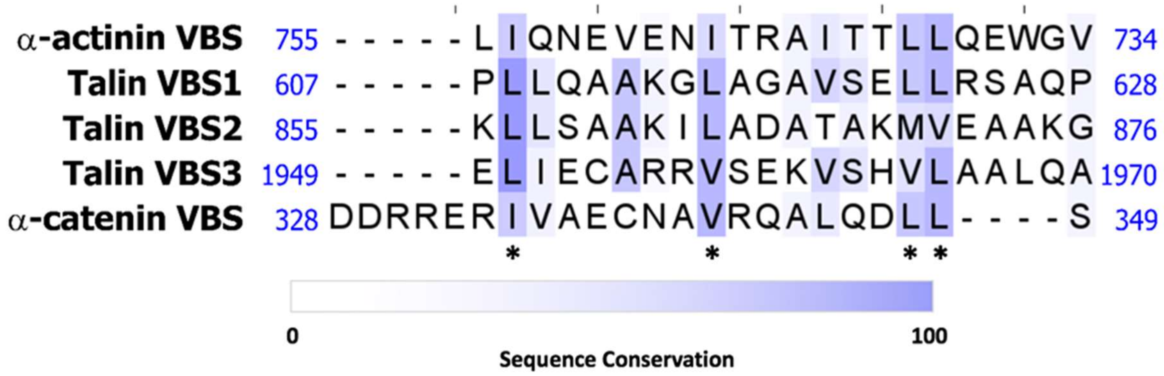

Supplementary Figure 1. Alignment of the amino acid sequences of α-actinin VBS (inverted), talin VBS1, VBS2, VBS3, and α-catenin VBS segments. The color schemes represent the sequence conservation within the alignment. Residues matching the consensus sequence are highlighted in dark blue. Residues that, while not matching the consensus, maintain a positive Blosum62 score (conservative substitution)[1] are shown in light blue. Non-matching residues and gaps in the sequence are depicted in white. The conserved residues marked with asterisks correspond to amino acids partially matching the canonical motif of the VBS sequence LxxAAxxVAxxVxxLIxxA previously reported by Izard T. and coworkers for VBS1, VBS2 and VBS3[2].

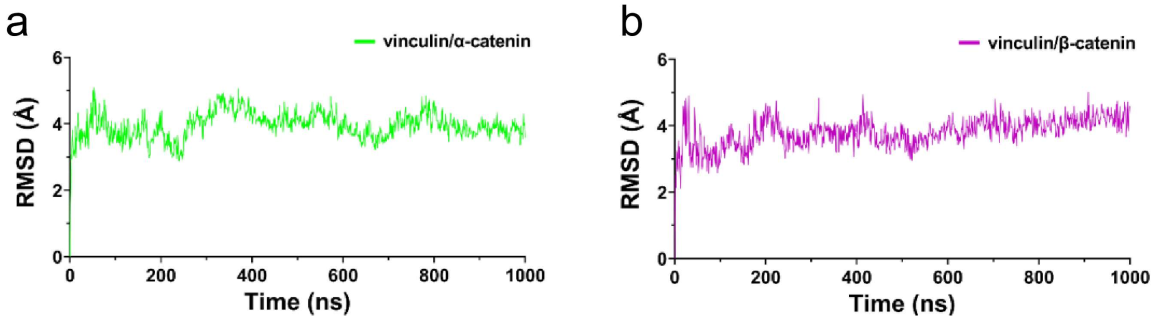

Supplementary Figure 2. Backbone roots mean standard deviation (RMSD) variation during 1 μs of MD simulation for vinculin/α-catenin (**a**) and vinculin/β-catenin (**b**) complexes.

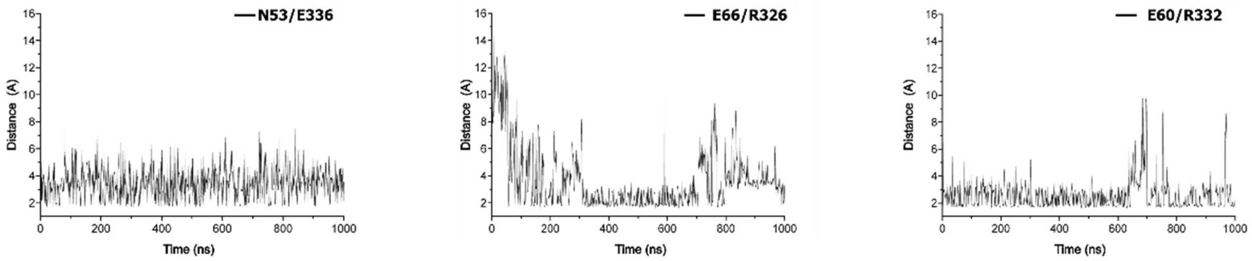

Supplementary Figure 3. Distance (Å) fluctuation plots of vinculin/α-catenin complex main interactions through 1 μs of MD simulation.

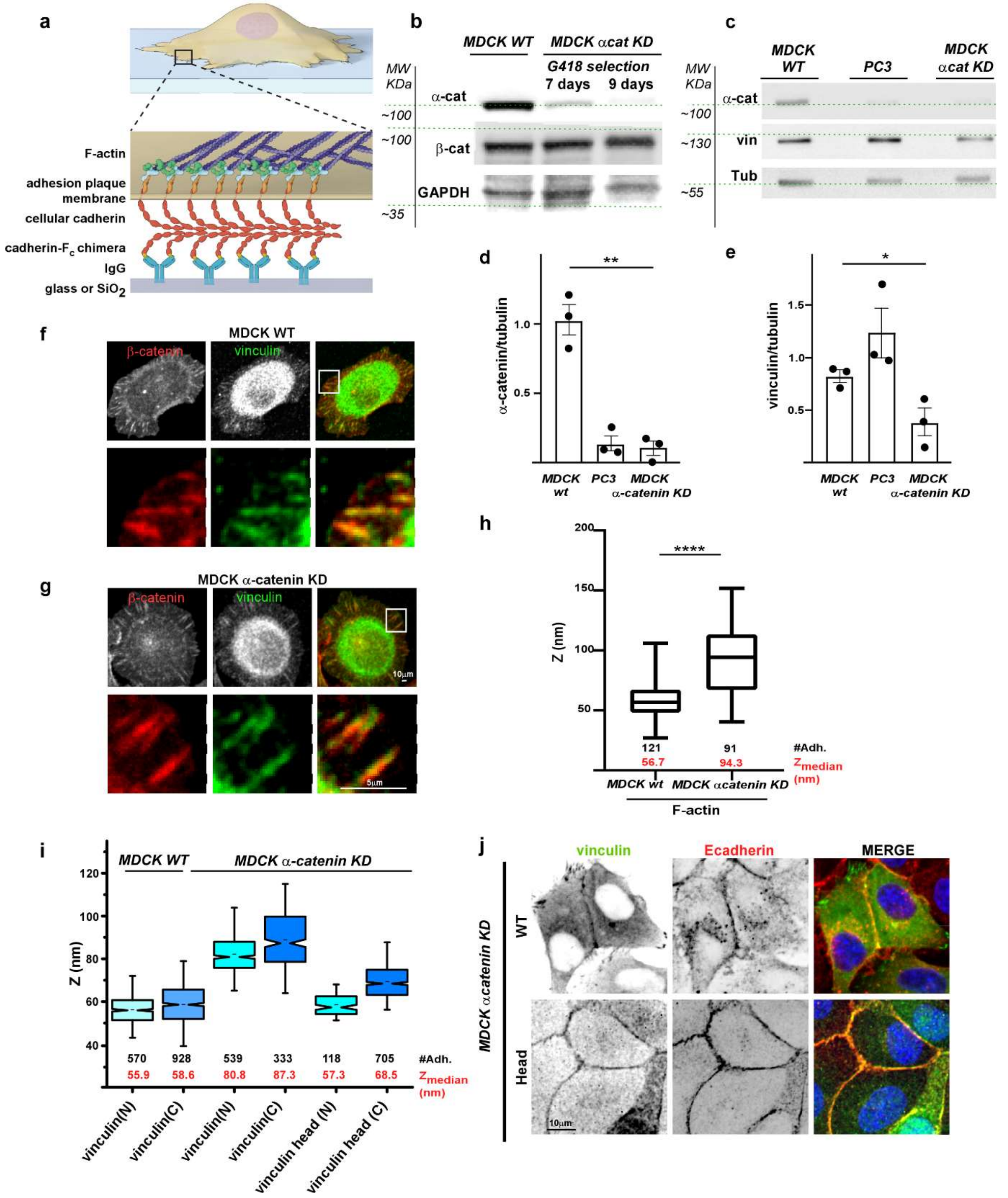

Supplementary Figure 4. (**a**) Schematic diagram of cadherin biomimetic substrate for high-resolution imaging of cadherin-based adhesions. Imaging substrate (grey) is coated by IgG (blue) and a chimera of F_c and E-cadherin extracellular domains (red) to support cell adhesions by cadherin homophilic interactions. The assets (molecules/cells/devices) were created by using Blender, a free 3D modeling and animation software (https://www.blender.org/) and the layout was made in Adobe Illustrator. (**b**) Representative western blot for α-catenin and β-catenin in MDCK α-catenin KD cells, at different days of selection with G410 (1mg/ml). MDCK wt are used as positive control. (**c**) Representative western

blots for vinculin and α-catenin in MDCK wt (positive control), PC3 (negative control) and MDCK α-catenin KD cells. (**d-e**) Plot histograms of quantification of (**d**) α-catenin (two tailed unpaired t-test for MDCK WT vs: MDCK α-catenin KD p=0.002, PC3 p=0.021) and (**e**) vinculin (two tailed unpaired t-test for MDCK WT vs: MDCK α-catenin KD p=0.041, PC3 p=0.164) expression levels in MDCK wt (positive control), PC3 (negative control) and MDCK α-catenin KD cells (n=3). (**f-g**) Immunofluorescence micrographs probing for endogenous β-catenin and vinculin in control MDCK (**f**) and MDCK αCat KD (**G**) seeded on cadherin biomimetic substrate as in panel **a** - bottom panels correspond to insets in top panels. Scale bars, 10 µm (top), 5 µm (bottom). (**h**) Notched box and whisker plots indicating the median $z$centre position of F-Actin in MDCK wt and α-catenin KD MDCK, obtained by iPALM imaging, as indicated in Figure **1j-k**. (**i**) Notched box and whisker plots indicating the median $z$centre position of vinculin wt and vinculin mutants in MDCK wt and α-catenin KD MDCK, as in Figure **2a**, but with vinculin head mutant. Box represents median, 1st, and 3rd quartiles; whiskers, 5th and 95th percentiles. (**j**) Localization of vinculin constructs wt and vinculin Head at AJs in MDCK α-catenin KD in monolayer. Maximum intensity projections of vinculin (GFP, green channel) and E-cadherin (antibody probes, red channel) as inverted grey scale images, and merged image. Scale bar, 10 µm.

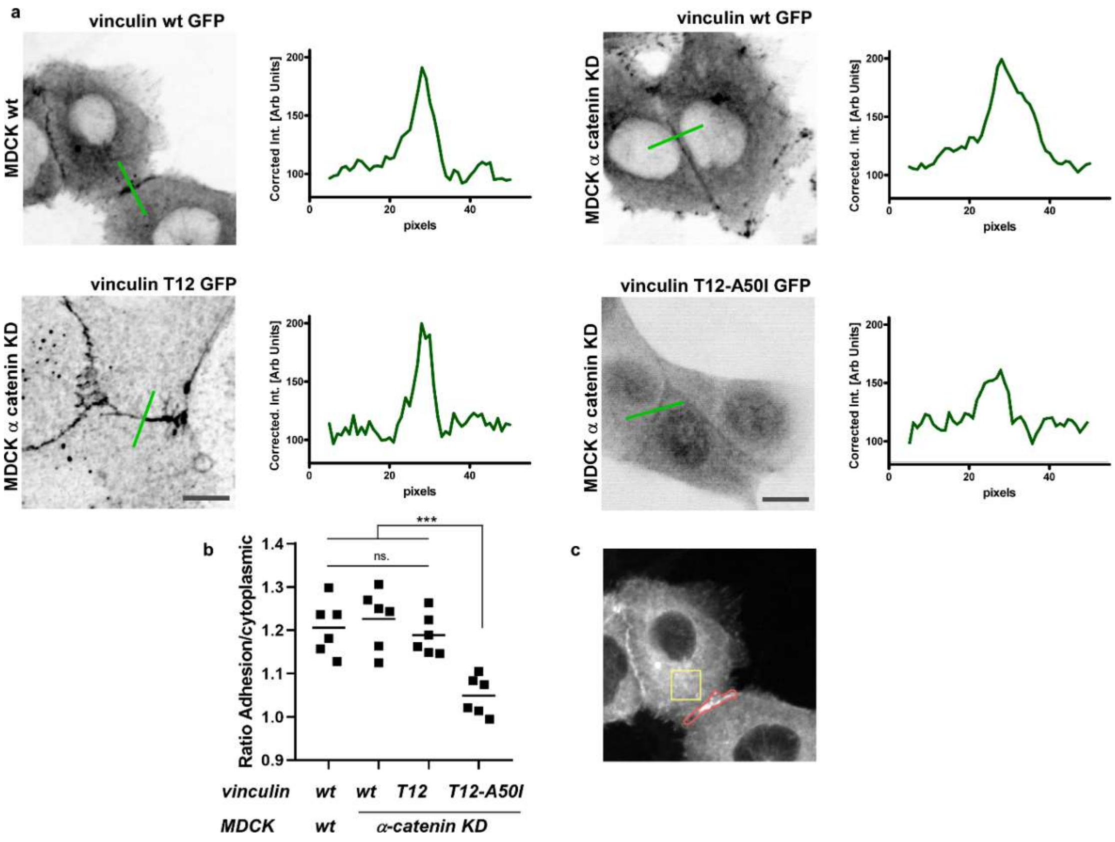

Supplementary Figure 5. (**a**) Line profiles for vinculin in MDCK WT and MDCK α-catenin KD as in Figure 2B and in Figure 3B to highlight the localization of vinculin constructs (wt, vinculin T12 and vinculin T12-A50I mutants) at cell-cell adhesions. Left, images for vinculin constructs transfected in MDCK wt and MDCK α-catenin KD are reported using inverted grey scale to enhance the contrast and highlight the localization at the adhesions. Scale bar 10um. Right, line profiles for green lines in the images denote specific localization of vinculin constructs at cell-cell adhesions. (**b**) Quantification of vinculin recruitment at cell-cell adhesion for each vinculin construct transfected in MDCK wt or MDCK α-

catenin KD (n=6). (**c**) Method used for the quantification in (**b**): ratio between cell adhesion intensity (red outline) and overall cell intensity (yellow box).

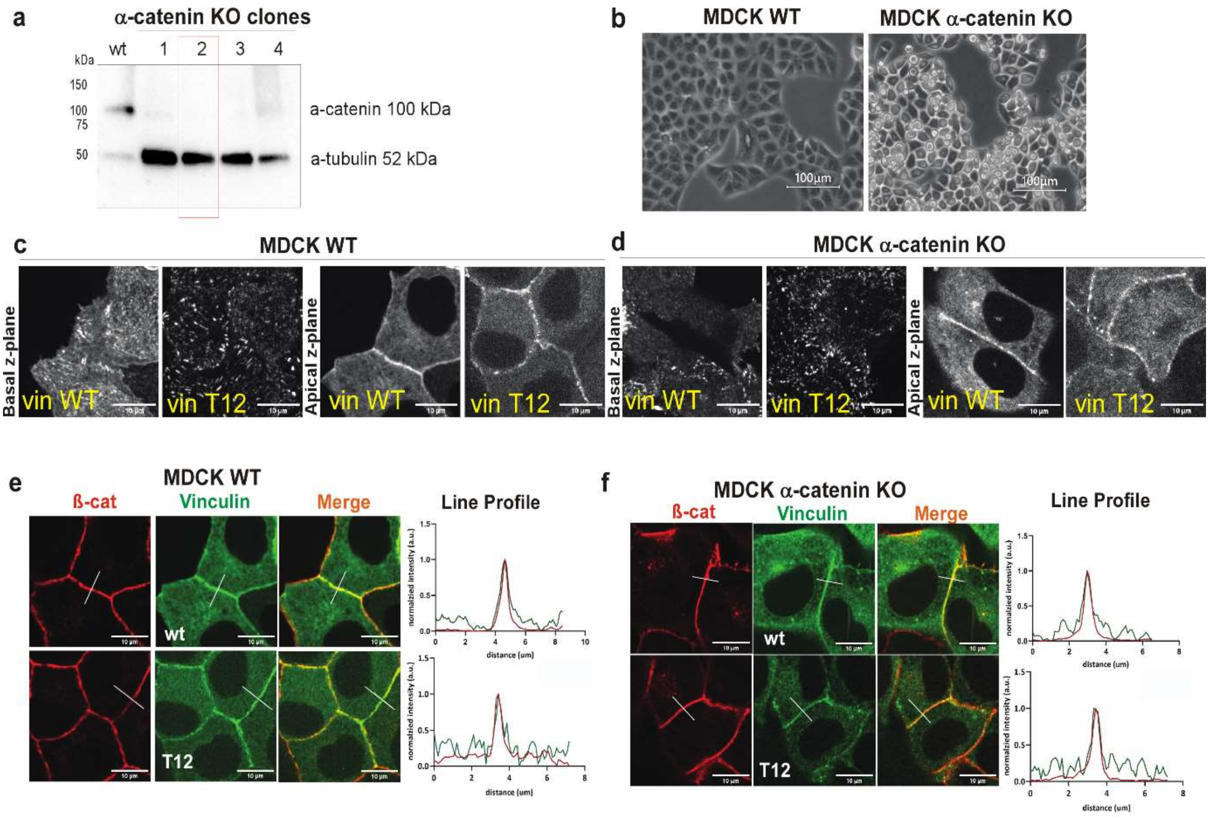

Supplementary Figure 6. Junctional co-localization of β-catenin and vinculin in α-catenin KO MDCK a-b) CRISPR/Cas9-mediated KO of α -catenin. (**a**) Immunoblot of α -catenin. Lysates of parental MDCK and α -catenin KO were probed for α -catenin with α -tubulin as loading control. Clones 2 used in the experiments are highlighted. (**b**) Phase contrast micrograph of parental MDCK (top) and α -catenin KO MDCK (bottom). (**c**) Localization of vinculin-wt or vinculin-T12 at cell adhesions of wt or α-catenin KO MDCK under control condition. (**d**-**f**) Immunofluorescence micrograph and line profiles, staining for endogenous β-catenin and vinculin constructs in parental MDCK or α -catenin KO MDCK. Comparison of Vinculin-wt and Vinculin-T12. Vinculin WT and vinculin-T12 mutant co-localize with β -catenin at cell-cell junctions in both parental MDCK as well as α -catenin KO. Scale bars: 100 µm for panel **b** and 10 µm for the remaining panels.

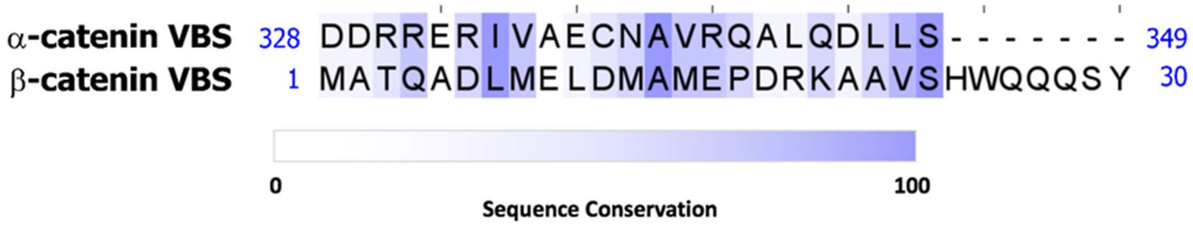

Supplementary Figure 7. Alignment of the α-catenin VBS and β-catenin VBS sequences. The color schemes represent the sequence conservation within the alignment. Residues matching the consensus sequence are highlighted in dark blue. Residues that do not match the consensus but still

maintain a positive Blosum62 score are shown in light blue. Non-matching residues and gaps in the sequence are depicted in white.

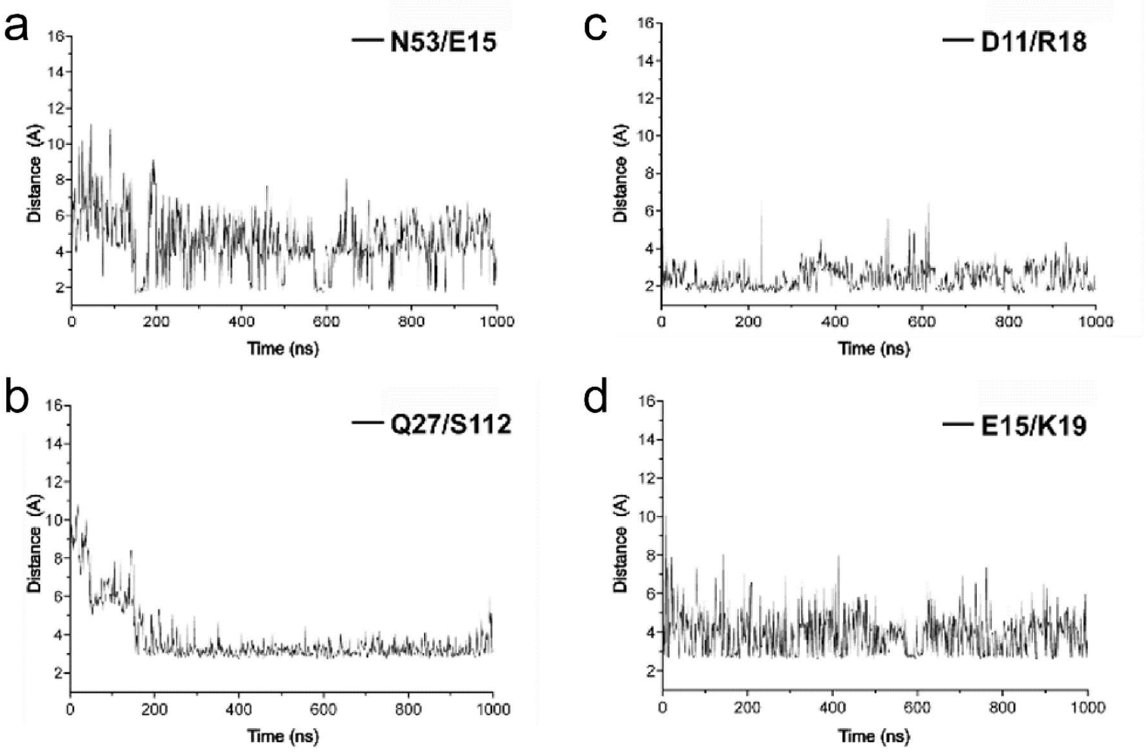

Supplementary Figure 8. (**a-b**) Fluctuation of the distance (Å) of intermolecular interactions observed in the MD simulation of the vinculin/β-catenin complex between residues N53/E15 and Q27/S112. (**c-d**) Fluctuation of the distance (Å) of intramolecular interactions of β-catenin observed in the MD simulation of the vinculin/β-catenin complex between residues D11/R18 and E15/K19.

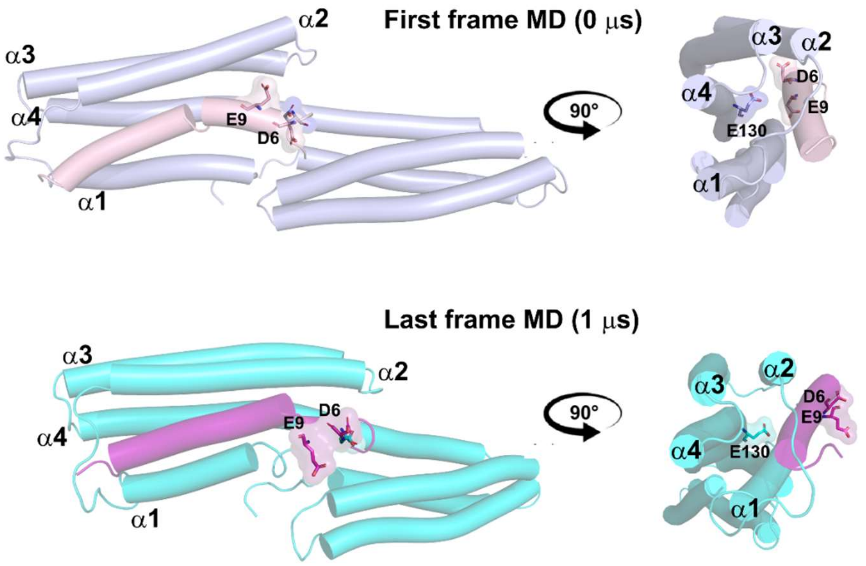

Supplementary Figure 9. Cartoon representation of vinculin/β-catenin complex in the first frame (0 μs, top) and after MD simulation (1 μs, bottom). Movement of the residues E130 of vinculin and D6 and E9 of β-catenin is highlighted to show coulombic repulsions.

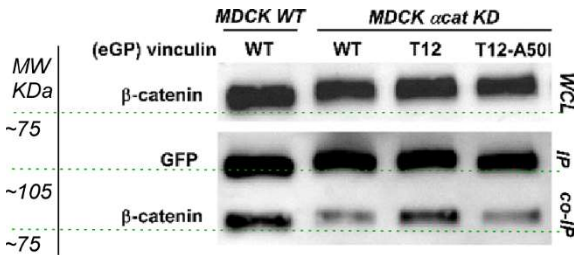

Supplementary Figure 10. Co-immunoprecipitation of β-catenin with vinculin wt and mutants. Representative western blots of three independent experiments (n=3) as in Figure 2C, probing for β-catenin pulled-down with GFP-vinculin constructs, including vinculin T12-A50I probed in MDCk wt and MDCKa-catenin KD cellstransfected with GFP-conjugated vinculin mutants. First row shows the whole cell lysate (WCL) as control.

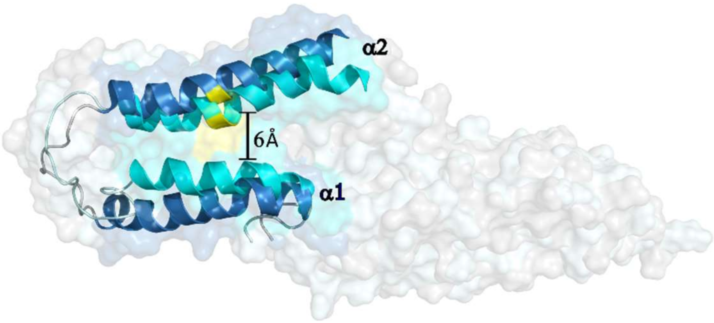

Supplementary Figure 11. Cartoon representation of vinculin(A50I) α-helix 1 (α1) and α-helix 2 (α2) from MD simulation of vinculin(A50I)/β-catenin complex. Two snapshots of the 1 μs MD is shown, the first frame is showed in blue, and the last frame is shown in cyan. The distance between α1 and α2 goes to ~12 Å at ~6 Å.

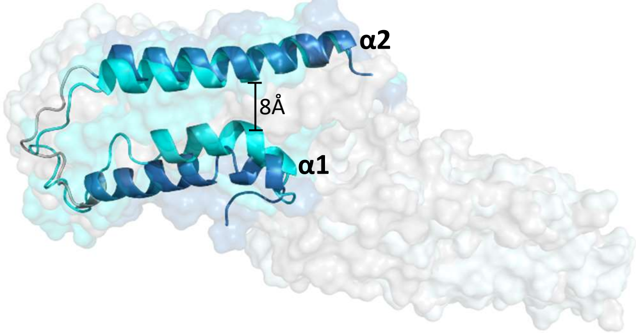

Supplementary Figure 12. Cartoon representation of vinculin α-helix 1 (α1) and α-helix 2 (α2) from MD simulation of vinculin/β-catenin(M8P) complex. Two snapshots of the 1 μs MD are shown: Dark blue indicate for the first frame, and cyan the last.

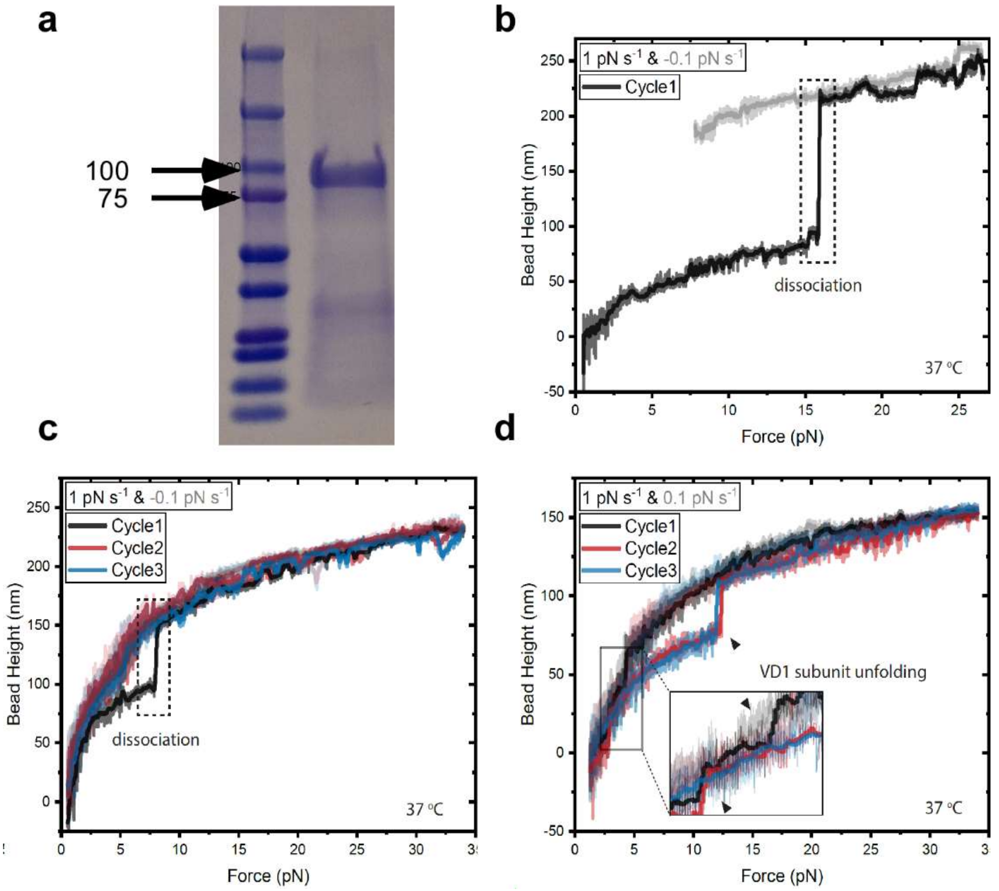

Supplementary Figure 13. (**a**) Coomassie blue stained gradient SDS-PAGE gel of β-catenin VBS-FH1-VD1 protein. Left lane: protein ladder (BIO-RAD #1610374). Right lane: elute protein was loaded onto a gradient SDS-PAGE gel (4-20%) and stained with Coomassie Blue. Molecular weight markers (100 and 75 kDa) are indicated on the left side of the gel. (**b**-**c**-**d**) Representative data from single protein manipulation experiments by vertical magnetic tweezer demonstrate the variety of ΔH observed. Force-dependent bead height change of VBS-VD1 detectors during force cycles with force increase at a loading rate of 1 pN/s (deep lines) and decrease at a loading rate of -0.1 pN/s (light lines). Experiments were performed at 37 ± 2 °C. (**b**) Significant step size of ~120 nm at ~16 pN indicated VBS-VD1 rupture and VD1 unfolding and (**c**) a ~52 nm step at 8 pN indicated VBS-VD1 dissociation and VD1 partially unfolding. (**d**) emonstrate an experiment where β-catenin VBS did not interact with VD1. Only small steps (< 35 nm) attributable to VD1 subunits unfolding is observed at ~ 12 pN. This last was observed in the majority of cases due to the comparatively low β-catenin VBS-VD1 binding energy.

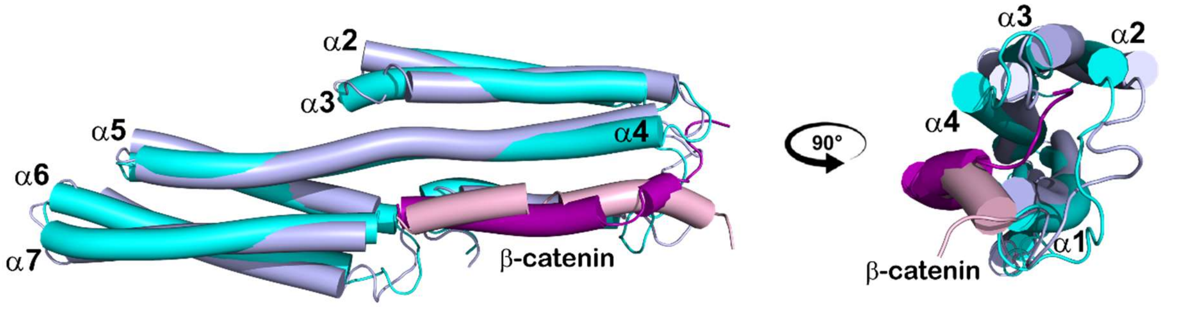

Supplementary Figure 14. Cartoon representation of vinculin (pale blue)/β-catenin (pink) complex (from trimer) at 0 ns of MD simulation and vinculin (cyan)/β-catenin (purple) complex at 500 ns of MD simulation.

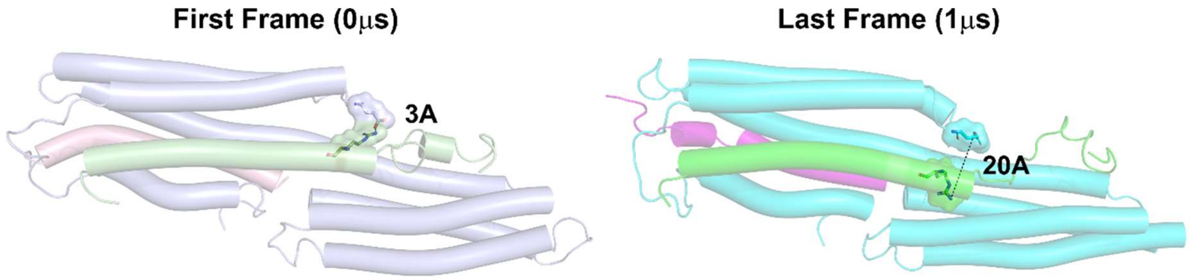

Supplementary Figure 15. Cartoon representation of vinculin/α-catenin/β-catenin complex at 0ns (left) and 1 μs (right) of MD simulation. The sticks show the residues R326 of α-catenin VBS and E60 of vinculin D1, which moves from 3 Å to a distance greater than 20 Å.

SUPPLEMENTARY REFERENCES

1. Eddy, S. R. Where did the BLOSUM62 alignment score matrix come from? *Nat Biotechnol* **22**, 1035–1036 (2004).
2. Izard, T. & Vonrhein, C. Structural Basis for Amplifying Vinculin Activation by Talin. *Journal of Biological Chemistry* **279**, 27667–27678 (2004).

SUPPLEMENTARY TABLES

Supplementary Table 1: Statistics of Protein Z-position as measured by surface-generated structured illumination microscopy. The position of fluorophores at the N- or C- termini are indicated.

Probes	cell type	z_{centre} (median, nm)	N_{Adh}	Mean (nm)	Std.dev (nm)
E-cadherin (N)	MDCK	46.56	352	49.95	4.7
β-catenin (N)	MDCK	52.44	334	52.62	4.6
β-catenin (C)	MDCK	49.70	969	50.85	7.3
α-catenin (N)	MDCK	38.97	517	39.39	5.44
α-catenin (C)	MDCK	51.95	988	51.67	7.09
Vinculin (N)	MDCK	55.93	570	56.18	6.44
Vinculin (C)	MDCK	58.61	928	58.66	8.91
F-Actin	MDCK	77.60	213	78.18	7.20
E-cadherin (N)	MDCK αcat KD	50.57	478	51.03	4.61
β-catenin (N)	MDCK αcat KD	51.63	404	52.18	5.65
β-catenin (C)	MDCK αcat KD	52.64	717	53.10	6.61
Vinculin (N)	MDCK αcat KD	80.82	539	82.15	8.70
Vinculin (C)	MDCK αcat KD	87.30	333	88.90	13.19
F-Actin	MDCK αcat KD	104.77	430	104.662	7.02
Vinculin head (N)	MDCK αcat KD	57.29	118	58.53	4.96
Vinculin TL-TS	MDCK αcat KD	68.45	705	69.42	7.62
Vinculin-T12 (N)	MDCK αcat KD	51.67	490	52.16	6.60
Vinculin-T12 (C)	MDCK αcat KD	82.30	309	83.44	11.99
Vinculin-T12 A50I (N)	MDCK αcat KD	69.8	168	69.88	5.82

Supplementary Table 2: Statistical analysis of Vinculin Z-position as measured by surface-generated structured illumination microscopy for the values in **Figure 1** and **Supplementary Table 1**. The position of fluorophores at the N- or C- termini are indicated.

Probes	cell type	Mean (nm)		Probes	cell type	Mean (nm)	P value t-test*	P value ANOVA**	Difference between means
Vinculin (N)	MDCK	56.18	vs.	Vinculin (C)	MDCK	58.66	n.a.	n.a.	2.480
Vinculin (N)	MDCK	56.18	vs.	Vinculin (N)	MDCK αcat KD	82.15	<0.0001	<0.0001	25.97
Vinculin (C)	MDCK	58.66	vs.	Vinculin (C)	MDCK αcat KD	88.90	<0.0001	<0.0001	30.24
Vinculin (N)	MDCK αcat KD	82.15	vs.	Vinculin (C)	MDCK αcat KD	88.90	n.a.	n.a.	6.757
Vinculin (N)	MDCK αcat KD	82.15	vs.	Vinculin-T12 (N)	MDCK αcat KD	52.16	<0.0001	<0.0001	29.98
Vinculin (C)	MDCK αcat KD	58.66	vs.	Vinculin-T12 (C)	MDCK αcat KD	83.44	n.a.	n.a.	5.464
Vinculin-T12 (N)	MDCK αcat KD	52.16	vs.	Vinculin-T12 (C)	MDCK αcat KD	83.44	<0.0001	<0.0001	31.28
Vinculin-T12 (N)	MDCK αcat KD	52.16	vs.	Vinculin-T12-A50I (N)	MDCK αcat KD	69.88	<0.0001	<0.0001	17.72

*T- Test nonparametric Kolmogorov Smirnoff
** Brown-Forsythe and Welch ANOVA test
n.a. difference between means (last column) is below the z-localization precision of SAIM (<9nm)

Supplementary Table 3: Statistics of cell-cell junction tension measurements by laser nanoscissor (**Figure 4**).

	n	Mean Recoil Rate (µm/s)	Std. dev. (µm/s)	s.e.m (µm/s)
MDCK ctrl	15	1.77	0.198	0.0511
MDCK αcat KD	13	1.34	0.264	0.0733
MDCK αcat KD + T12	14	1.88	0.451	0.121

Supplementary Table 4: Statistical analysis of cell-cell junction tension measurements by laser nanoscissor experiments in **Figure 4** and **Supplementary Table 3**.

Probes	cell type		Probes	cell type	P value ANOVA*
Vinculin wt	MDCK wt	Vs	Vinculin wt	MDCK αcat KD	0.0030
Vinculin wt	MDCK αcat KD	Vs	Vinculin T12	MDCK αcat KD	0.0003
Vinculin wt	MDCK wt	Vs	Vinculin T12	MDCK αcat KD	n.s.

*Ordinary one-way ANOVA with Sidak correction for multiple comparisons.

Supplementary Table 5: Statistics of FRAP measurements (**Figure 4**). Fit of FRAP curves are fitted to a single exponential function, $I(t) = p(1-e^{-kt})$. Half-time is $ln2/k$.

	n	p, mobility fraction			k (s^{-1}), rate of diffusion			$t_{1/2}$ (s), half-time	
		Mean	Std. dev.	s.e.m.	Mean	Std. dev.	s.e.m.	Mean	Std. dev.
MDCK ctrl	26	0.331	0.0676	0.0133	0.0271	0.0111	0.0022	29.02	9,896
MDCK αCat KD	31	0.553	0.177	0.0318	0.0356	0.0163	0.0029	24.03	11.02
MDCK αCat KD + Vinculin-T12	19	0.402	0.0718	0.0165	0.0377	0.0121	0.0029	21.10	9.790
MDCK αCat KD + Vinculin head	6	0,598	0.112	0,0455	0,0372	0,0121	0,0049	20.36	6.706

Supplementary Table 6: Statistical analysis of p, mobility fraction of FRAP measurements in **Figure 4** and **Supplementary Table 5**.

Probes	cell type		Probes	cell type	P value ANOVA*
Vinculin wt	MDCK wt	Vs	Vinculin wt	MDCK αcat KD	<0.0001
Vinculin wt	MDCK αcat KD	Vs	Vinculin T12	MDCK αcat KD	<0.0001
Vinculin wt	MDCK wt	Vs	Vinculin T12	MDCK αcat KD	n.s.

*Ordinary one-way ANOVA without correction for multiple comparisons

Supplementary Table 7: Statistical analysis of wound-model assay of **Figure 4i-j**, **Supplementary Video 9-10-11** indicating high correspondence between mode of migration of MDCK wt and MDCK α-catenin KD transfected with vinculin T12 mutant. Mean of the major angular component of the frequency distribution and relative angular speed magnitude as well as their respective statistical analysis (p values) are calculated by performing a Gaussian fitting of the angular distributions of the single experiments.

ANOVA comparison of fitted major angular component	WT mean	α-cat KD mean	α-cat T12 mean	WT vs α-cat KD p value	α-cat KD vs α-cat T12 p value	WT vs α-cat T12 p value
Frequency	-11.13 °	7.24 °	-5.6 °	0.17	0.36	0.81
Relative angular speed magnitude	-9.61 °	6.21 °	-7.3 °	0.27	0.37	0.97

Supplementary Table 8: Main residues that participate in the generation of inter and intra molecular interactions in the stabilization of the complex throughout molecular dynamics simulation.

DIMER			
	Vinculin D1	α-catenin VBS	
Intermolecular Interaction	Q19	S349	
	N53	E336	
	E60	R332	
	E60	R329	
	E66	R326	
	R105	T351	
	K170	S323	
	Vinculin A50I D1	α-catenin VBS	
Intermolecular Interaction	N53	E336	
	E60	R332	
	Q19	S349	
	E181	R330	
Hydrophobic Interaction	V137	L318	
	I141		
	Vinculin D1	β-catenin VBS	
Intermolecular Interaction	N53	E15	
	S112	Q27	
Intramolecular interaction		K19	E15
		R18	E15
		R18	D11
	Vinculin A50I D1	β-catenin VBS	
Intermolecular Interaction	R7	D6	
Intramolecular interaction		K19	E15
		R18	E15
		R18	D11
TRIMER			
	Vinculin D1	α-catenin VBS	β-catenin VBS
Intermolecular Interaction	M26	Y351	W25
Intramolecular Interaction	N53	E336	

Supplementary Table 9: Statistical analysis of free energies (ΔG) of data from **Supplementary Data 1**. P values for the individual comparisons are indicated. In the text, ΔG values are considered to be statically significantly different for p < 0.05).

Dimers	Mean ΔG (kcal/mole)	SD	number of timesteps		Comparison with Vinculin/ α-catenin (P value ANOVA*)
Vinculin/ α-catenin	-63,1755	9,5759	100		
Vinculin(A50I)/ α-catenin	-66,0113	13,1273	100	vs	0,0927
Vinculin/ α-catenin(L344P)	-34,3468	7,1111	100	vs	<0,0001
					Comparison with Vinculin/ ß-catenin (P value ANOVA*)
Vinculin/ß-catenin	-28,9015	6,7309	100		
Vinculin(A50I)/ ß-catenin	-25,4896	7,3484	100	vs	0,0028
Vinculin/ ß-catenin(M8P)	-25,6784	8,3282	100	vs	0,0050

*Ordinary Anova with Dunnet comparison.
