# Supplementary material for: Alternative molecular mechanisms for force transmission at adherens junctions via β-catenin-vinculin interaction
Source: Nat Commun. 2024 Jul 5;15:5608. doi: 10.1038/s41467-024-49850-5 (PMC11226457; doi:10.1038/s41467-024-49850-5)
Supplement: Supplementary file 3 — Description of Additional Supplementary Files [file 41467_2024_49850_MOESM3_ESM.pdf]

Description of Additional Supplementary Files:

Supplementary Data 1: α-C-backbone RMSD: values for each complex along 1μs of MD simulation. Data were extracted every 100 ns.

Supplementary Data 2: Decompose of binding free energy for each complex obtained through MM-PBSA method: The energy component corresponds to the following: van der Waals (VDWAALS), electrostatic energy (EEL), electrostatic contribution calculated by Poison Bolztman (EPB), nonpolar energy calculated by an empirical model (ENPOLAR), dispersion energy (EDISPER), delta gas phase energy (DELTA G gas) and delta solvate phase energy (DELTA G solv). The final estimated binding free energy (DELTA TOTAL) calculated from the terms above. All energy terms are expressed in kcal/mol.

Supplementary Movie 1: Molecular dynamics simulation of the dimer vinculin D1 domain and α-catenin VBS segment. Graphic video of the trajectory, for the duration of 1 μs total time, for vinculin D1 domain (cyan) and α-catenin VBS segment (green) complex, produced using VMD 1.9.3 software.

Supplementary Movie 2: Molecular dynamics simulation of the dimer vinculin D1 domain and β-catenin VBS segment. Graphic video of the trajectory, for the duration of 1 μs total time, for vinculin D1 domain (cyan) and ⍰-catenin VBS segment (purple) complex, produced using VMD 1.9.3 software.

Supplementary Movie 3: Molecular dynamics simulation of the dimer vinculin (A50I) D1 domain and β-catenin VBS segment. Graphic video of the trajectory, for the duration of 1 μs total time, for vinculin (A50I) D1 domain (cyan) and ⍰-catenin VBS segment (purple) complex, produced using VMD 1.9.3 software.

Supplementary Movie 4: Molecular dynamics simulation of the dimer vinculin (A50I) D1 domain and α-catenin VBS segment. Graphic video of the trajectory, for the duration of 1 μs total time, for vinculin (A50I) D1 domain (cyan) and α-catenin VBS segment (green) complex, produced using VMD 1.9.3 software.

Supplementary Movie 5: Molecular dynamics simulation of the dimer vinculin D1 domain and α-catenin (L344P) VBS segment. Graphic video of the trajectory, for the duration of 1 μs total time, for vinculin D1 domain (cyan) and α-catenin (L344P) VBS segment (green) complex, produced using VMD 1.9.3 software.

Supplementary Movie 6: Molecular dynamics simulation of the dimer vinculin D1 domain and β-catenin (M8P) VBS segment. Graphic video of the trajectory, for the duration of 1 μs total time, for vinculin D1 domain (cyan) and β-catenin (M8P) VBS segment (purple) complex, produced using VMD 1.9.3 software.

Supplementary Movie 7: Measurement of junctional tension by laser ablation. Representative video showing elastic recoil upon laser ablation for each condition indicated in Figure 4C. MDCK cells (wt and α-catenin KD) were co-transfected as indicated (vinculin or vinculin T12). ZO-1 mEmerald marks the cell-cell junctions. Imaging was performed using Nikon A1R MP laser scanning confocal microscopy equipped with a CFI Plan Apo 60X NA 1.45 oil immersion objective. Images were acquired every 2 s, starting from 6 s before ablation and for 30 s after it. For details, please refer to the Methods section.

Supplementary Movie 8: Measurement of protein turnover at AJ by FRAP. Representative video of FRAP for each condition indicated in Figure 4F. MDCK cells (wt and α-catenin KD) were transfected with the indicated constructs (vinculin GFP or vinculin T12 GFP). Imaging was performed using a Nikon Eclipse Ti-E inverted microscope with a CFI Plan Apo 60X NA 1.45 oil immersion objective. Images were acquired every 2 s. For details, please refer to the Methods section.

Supplementary Movie 9: Wound-model migration assay and PIV analysis of MDCK cells wt were transfected with vinculin GFP. Videos show the transmission imaging of cell migration (left) and the corresponding PIV (particle image velocimetry) quiver over-imposed on transmission image (Right). Vectors indicate direction and magnitude of movement. Vector length and rainbow color coding are used to highlight difference in speed. Imaging was performed using a BioStation IM-Q imaging system (Nikon) with a CFI Plan Apo 20X NA 0.5 objective. Images were acquired every 10 minutes. For details, please refer to the Methods section.

Supplementary Movie 10: Wound-model migration assay and PIV analysis of MDCK α-catenin KD with vinculin GFP. Videos show the transmission imaging of cell migration (left) and the corresponding PIV (particle image velocimetry) quiver over-imposed on transmission image (Right). Vectors indicate direction and magnitude of movement. Vector length and rainbow color coding are used to highlight difference in speed. Imaging was performed using a BioStation IM-Q imaging system (Nikon) with a CFI Plan Apo 20X NA 0.5 objective. Images were acquired every 10 minutes. For details, please refer to the Methods section.

Supplementary Movie 11: Wound-model migration assay and PIV analysis of MDCK α-catenin KD with vinculin GFP. Videos show the transmission imaging of cell migration (left) and the corresponding PIV (particle image velocimetry) quiver over-imposed on transmission image (Right). Vectors indicate direction and magnitude of movement. Vector length and rainbow color coding are used to highlight difference in speed. Imaging was performed using a BioStation IM-

Q imaging system (Nikon) with a CFI Plan Apo 20X NA 0.5 objective. Images were acquired every 10 minutes. For details, please refer to the Methods section.

Supplementary Movie 12: Wound-model migration assay for PC3 cells. Representative video of migration assay of PC3 cells, a prostatic cancer cell line that lacks α-catenin (Supplementary Figure 4C-D for expression levels of α-catenin) but still can migrate collectively. Imaging have been performed with Muvicyte (Perkin Elemer), objective Olympus LMPlanFL N, 20x NA0.40. Images were acquired every 20 min for a total time of 48 h and 44 min.

Supplementary Movie 13: Molecular dynamics simulation of the trimer vinculin D1 domain, βcatenin VBS and α-catenin VBS segment. Graphic video of the trajectory, for the duration of 1 μs total time, for the trimer vinculin D1 domain (cyan), β-catenin VBS (purple) and α-catenin VBS segment (green), produced using VMD 1.9.3 software. View of β-catenin VBS (purple) on S3 binding pocket.

Supplementary Movie 14: Molecular dynamics simulation of the trimer vinculin D1 domain, βcatenin VBS and α-catenin VBS segment. Graphic video of the trajectory, for the duration of 1 μs total time, for the trimer vinculin D1 domain (cyan), β-catenin VBS (purple) and α-catenin VBS segment (green), produced using VMD 1.9.3 software. View of α-catenin VBS (green) on S1 binding pocket.
